# Louisiana

**Published:** 1896-11

**Authors:** 


*Louisiana.* Tuberculosis has been found among the herd of the State Experiment Station, a test revealing six of the herd of twenty-two affected. These latter have been isolated, and, with the healthy ones, will be retested from time to time and the diseased ones returned to the herd only when they cease to react. The calves will be taken from the mothers at birth and fed milk from healthy cows only, to see how thoroughly the disease may be eradicated by this system (Prof. Bang's), and thus avoid the great pecuniary loss to the station. All of the cattle responding were well bred, and among those purchased native cattle seem to have a greater immunity from the disease. A test of the relative percentage of butter-fat present in some nine of the animals before and after injection proved interesting. The variations were quite marked, in some instances as much as 2.8 per cent. But one cow, a Jersey, presenting generalized tuberculosis, was destroyed, and the carcass cremated. Disinfection of premises will be carried on at regular intervals.

